# Trial-level ERPs predicted behavioral responses during self-referential processing in late childhood

**DOI:** 10.1093/scan/nsae011

**Published:** 2024-02-02

**Authors:** Pan Liu, Xiao Yang, Jaron X Y Tan

**Affiliations:** Department of Psychology, University of Alberta, Edmonton, AB T6G 2E9, Canada; Departments of Health Sciences and Computer Sciences, Northeastern University, Boston, MA 02115, USA; Department of Psychology, North Dakota State University, Fargo, ND 58102, USA

**Keywords:** self-schematic processing, EEG, multilevel model, trial-level analysis, pre-adolescence

## Abstract

Self-referential information is uniquely salient and preferentially processed even in children. The literature has used the self-referent encoding task (SRET) combined with event-related potentials (ERPs) to study self-referential processing and its associations with youth psychopathology. However, it is unclear how the ERP and behavioral indices of SRET are associated with each other, although this knowledge can promote our mechanistic understanding of this construct and its role in psychopathology. We examined this question in 115 9- to 12-year-old children, a critical period for the development of self-related concepts. By applying a multilevel modeling approach to the trial-level data of SRET, we disaggregated the between- and within-person variability and observed within-person, but not between-person, effects of the P2 and late positive potential (LPP) on behavioral responses: a larger P2 on a given trial predicted a faster response in this trial; a larger LPP on a given trial predicted a higher likelihood of endorsing the word of this trial. We provided novel evidence on how the within-person variability of the ERPs predicted the overt responses of the SRET in children. These findings inform our mechanistic knowledge of self-referential processing and shed light on a better understanding of the role of self-referential processing in the development of psychopathology.

## Introduction

Across development, information that references or describes oneself (i.e. self-referential information) is uniquely salient and preferentially processed compared to other types of information even in children (Andrews *et al*., 2020; Pfeifer *et al*., 2007, 2016; Hudson *et al*., 2021). In examining the mechanisms of self-referential processing and its potential links with psychopathology, extant studies have used the established self-referent encoding task (SRET; Derry and Kuiper, 1981) to measure this construct in youths and adults. Other than behavioral indices, the SRET can be readily combined with neuroscience measures to tap the brain correlates of self-referential processing. Event-related potential (ERP), quantified as voltage fluctuations of scalp-recorded electroencephalogram (EEG) signals time-locked to ‘events’ such as stimulus onset, is an economical, child-friendly technique with high temporal resolution and good psychometric properties (Cassidy *et al*., 2012; Huffmeijer *et al*., 2014), particularly adept to characterize the temporal dynamics of information processing in children. In this literature, typically developing children show a ‘self-positivity bias’, i.e. a deeper processing of positive (*vs* negative) self-referential information, indexed by both the behavioral and ERP indices (Goldstein *et al*., 2015; Liu *et al*., 2020a,b; Hudson *et al*., 2021; Liu and Tan, 2023a,b). In contrast, a shallower processing of positive, or a deeper processing of negative, self-referential cues, is associated with clinical or subclinical depression in youths (Auerbach *et al*., 2015; Speed *et al*., 2016; Allison *et al*., 2021; Liu and Tan, 2023a,b).

However, it remains unclear to what extent the behavioral and ERP indices of the same construct—self-referential processing—are associated with each other. While a multi-measurement approach has been advocated to facilitate mechanistic research in psychopathology (Cuthbert & Insel, 2013), the lack of knowledge of how different measures can be integrated prevents a better understanding of the target construct and its role in psychopathology. The same issue exists for other cognitive constructs implicated in psychopathology (e.g. attentional bias, Wise *et al*., 2022). From an information processing perspective, the continuous flow model contends that the information of a (visual) stimulus is extracted and accumulated rapidly but continuously over time, during which a deeper evaluation of the stimulus unfolds until an overt behavioral response is executed (Coles *et al*., 1985, 1995). Based on this model, it is reasonable to expect that the earlier extraction and evaluation of self-referential information, which can be readily assessed by the ERPs, will predict the overt behavioral responses captured by the SRET. This study aimed to examine this question in a group of typically developing children of ages 9–12 years, a critical period for the development of self-concepts. Notably, the relevant ERP studies have used a between-person analytic approach that assumed within-person variations as noises. To address this limitation, we employed a novel multilevel modeling (MLM) approach to glean meaningful information from both the between- and within-person levels. This approach is particularly suitable to study children, given the vast inter- and intra-individual variations existing in their developing brain and behaviors (Mills *et al*., 2021).

### Behavioral and ERP measures of self-referential processing

While the SRET was initially established to measure adults’ self-referential processing (Derry and Kuiper, 1981; Kuiper and Derry, 1982), this paradigm has proved useful to study children and has been successfully applied in children as young as 6 years old (Goldstein *et al*., 2015). The SRET consists of a self-endorsement task followed by an unexpected memory task. During the self-endorsement, participants are presented with a series of positive and negative personal trait words and decide whether each word is self-descriptive (‘Does this word describe you?’). As common practice, the endorsement task is followed by an unexpected memory task (e.g. recall or recognize the presented words), which taps into a more in-depth or latent facet of self-referential processing (Northoff *et al*., 2006). In performing this task, participants generate a series of behavioral indices of self-referential processing, including the endorsement/rejection of each word, the response time (RT) in endorsing/rejecting each word and the recall/recognition of each word. Consistent with a ‘self-positivity bias’, typically developing children tend to endorse and recall (or recognize) more positive words but only a minimum number of negative words (e.g. Goldstein *et al*., 2015; Hudson *et al*., 2021; Liu *et al*., 2020a; Liu *et al.*, 2020b) they also show faster RTs in endorsing positive words or rejecting negative words but slower RTs in endorsing negative, or rejecting positive, words (Liu *et al*., 2020b; Hudson *et al*., 2021).

ERP studies in youths and adults have reported several ERP components elicited by the SRET, including the P1, P2 and late positive potential (LPP), each representing a different stage of self-referential processing. The P1 is a positive deflection peaking around 100–200 ms post-stimulus onset and maximizing at the parieto-occipital channels; it is thought to reflect early, fast visual attentional processing mediated by the visual cortex (Mangun & Buck, 1998; Hillyard *et al*., 1998). The P2 is a positive deflection that peaks around 200–300 ms post-stimulus onset and maximizes at fronto-central sites (Crowley and Colrain, 2004), reflecting more controlled attentional processes, such as sustained perceptual processing (Schupp *et al*., 2004) and the allocation and mobilization of attentional resources toward salient cues (Bar-Haim *et al*., 2005; Eldar *et al*., 2010). The LPP is a late, slow positive deflection that starts around 500–600 ms post-stimulus and lasts through stimulus presentation. Typically elicited by elevated stimulus salience, the LPP is thought to reflect a deeper, more elaborative processing of socio-emotional meanings that involves sustained engagement and memory retrieval (Cuthbert *et al*., 2000; Foti and Hajcak, 2008; Foti *et al*., 2009; Macnamara *et al*., 2009; Weinberg *et al*., 2012, 2021). While studies using non-referential emotion processing paradigms typically reported the LPP in posterior regions, many of the SRET studies have reported a more anterior LPP (aLPP) across midline fronto-central channels (e.g. Shestyuk and Deldin, 2010; Auerbach *et al*., 2015, 2016; Liu and Tan, 2023a,b). Some of these studies found that healthy youths showed a potentiated P1, P2 or aLPP toward the positive *vs* negative SRET words, indicating a deeper processing of positive *vs* negative self-referential information (Auerbach *et al*., 2016; Liu and Tan, 2023b). However, the posterior LPP (pLPP) did not differentiate between the positive and negative SRET conditions (Liu and Tan, 2023b), suggesting that during the SRET, the pLPP might reflect more generic socio-emotional information processing, while the aLPP might more specifically reflect self-related processes.

### Associations between the behavioral and ERP indices of self-referential processing

Although many studies have employed both the behavioral and ERP indices of the SRET to study self-referential processing, only a few have examined the associations between the two measures via between-person bivariate correlation. One study found that in healthy adolescents, a larger LPP toward positive (or negative) SRET words was correlated with a greater number of positive (or negative) words recalled (Auerbach *et al*., 2016); other studies did not report any significant correlations (Speed *et al*., 2016; Liu and Tan, 2023a,b). These inconsistent findings may be related to the different samples examined in these studies, including healthy female adolescents of ages 13–18 years (Auerbach *et al*., 2016), healthy children of ages 9–12 years (Liu and Tan, 2023a, 2023b) and adolescent girls of ages 8–14 years with a maternal history of depression (Speed *et al*., 2016). Further, these studies used bivariate correlation only, which failed to tap the nuanced patterns of relationships between variables. It is necessary to systematically examine the brain-behavior relationships of self-referential processing via more sophisticated methods.

### The present study

The extant literature has exclusively used a between-person analytic approach (including bivariate correlation) by averaging (or aggregating) the ERP or behavioral signal in each trial across all trials of a condition for each participant. Although signal averaging can increase the signal-to-noise ratio (Luck, 2014), this approach assumes that the signal is constant from trial to trial and that any within-person, trial-level variations are noises. Nonetheless, information processing is a dynamic process that fluctuates within even short time windows and can be impacted by factors such as habituation, sensitization and the features of a given stimulus (e.g. Woestenburg *et al*., 1983; Regtvoort *et al*., 2006). Trial-to-trial variations caused by these factors may reflect meaningful patterns of information processing that cannot be captured by between-person analysis. Indeed, psychological processes are often non-ergodic; the between-person, time-invarying effect (i.e. effect of differences in trait-like characteristics when comparing a person with another person) is rarely the same as the within-person, time-varying effect (i.e. effect of changes when comparing a person with themselves over time; Molenaar, 2004; Curran and Bauer, 2011). Isolating these two portions of the variations is therefore critical to fully understand the mechanisms of the cognitive process of interest.

An MLM approach can address this issue by modeling and disaggregating the between- and within-person sources of variability, which will elucidate the cognitive mechanisms of self-referential processing. This knowledge, in turn, may promote our understanding of the role of self-referential processing in psychopathology. The MLM approach has been successfully applied in the adult ERP literature (Volpert-Esmond *et al*., 2018, 2021; Von Gunten *et al*., 2018; Volpert-Esmond and Bartholow, 2021). For example, a study on face processing found that for an individual (i.e. on the within-person level), a larger P2 elicited by a given face predicted faster RT in categorizing this face; this effect was not found on the between-person level (Volpert-Esmond and Bartholow, 2021). Based on this literature, we expected that applying the MLM to the SRET data in children will similarly generate interesting findings of the within-person level brain-behavior linkages that cannot be captured by between-person analysis.

Data reported in this study were collected from 115 typically developing children of 9–12 years old, a critical developmental period for self-related constructs characteristic of substantial inter-individual and intra-individual variations. For the EPR indices, we included the P1, P2 and LPP elicited during the SRET; for the behavioral indices, we included the endorsement/rejection of each word, the RT in endorsing/rejecting each word and the recall of each word. Informed by the continuous flow model of information processing (i.e. information accumulates over time and determines subsequent overt responses; Coles *et al*., 1985, 1995), we were interested in how the SRET-elicited ERPs predicted children’s behavioral responses to the SRET stimuli. Based on previous findings from adults (Volpert-Esmond and Bartholow, 2021), we expected to observe differential patterns of the brain-behavior associations on the between- *vs* within-person levels. Given the known functions of the ERP components (e.g. Bar-Haim *et al*., 2005; Foti and Hajcak, 2008; Mangun & Buck, 1998), we speculated that the early components, P1 and P2, might be more closely associated with RT, whereas the LPP might be more closely associated with the endorsement and recall of the words. However, given the scarcity of MLM research on self-referential processing, we considered this study as exploratory without more specific hypotheses on the patterns of the associations. The focus of this study was to examine the relationships between different measures of self-referential processing rather than their associations with psychopathology (which will be the next step of our research); thus, we treated children’s concurrent depressive symptoms as a covariate, rather than an outcome, in the MLM.

## Method

### Participants and procedure

Data were drawn from a larger study examining the neurobehavioral correlates of socio-emotional processing in children. One hundred fifteen 9- to 12-year-old typically developing youths (66 girls, 49 boys; mean age/s.d. = 10.98/1.18 years) were recruited from the local communities in a Midwestern urban area in the USA. Children with reported major physical diseases, serious mental illness or neurodevelopmental disabilities were not recruited. The demographics of the sample lined up with the local demographics (87.5% White, 3.6% Asian, 8.9% multiracial and 7.2% Hispanic or Latino; family income range: $15 000–$350 000).

Children and caregivers were invited to a laboratory visit, where caregiver consent and youth assent were first acquired. Each child completed four EEG tasks (including the SRET) in a counterbalanced order and an eye-tracking task that measured different aspects of socio-emotional processing. Only the SRET data were presented here. Following the laboratory visit, children completed a questionnaire package assessing their socio-emotional functions via Qualtrics at home, including the 27-item Children’s Depression Inventory (CDI; Kovacs, 1978). Due to the limited variability, we excluded the item ‘I want to kill myself.’ For each of the remaining 26 items, children were asked to select one of the three options that best described them for the past 2 weeks (e.g. I am sad: 0 = once in a while, 1 = many times or 2 = all the time). A total score (ranging from 0 to 52) was calculated as an indicator of depressive symptoms (Cronbach’s *α* = 0.91). Participants received monetary compensation upon completing the study. The protocol was approved by the institutional review board of the university.

### The SRET paradigm

The SRET paradigm consisted of 60 personal trait words (30 positive and 30 negative) adopted from previous research on youths of similar ages (e.g. Speed *et al*., 2016). The positive and negative words differed in valence but were matched on arousal and length based on the Affective Norms for English (Bradley and Lang, 1999). The 60 words were presented in a pseudo-random manner, with no more than two words of the same valence presented in a row. Each trial started with a 500 ms fixation cross, after which a positive or negative word was presented for 1000 ms, followed by another 500 ms fixation cross. Next, a question (‘Does this word describe you?’) popped up until children pressed one of the two buttons on a response box to indicate their answers (left button = yes and right button = no). Immediately following the endorsement task, youths were unexpectedly asked to recall as many of the presented words as possible for up to 2 minutes. We recorded behavioral data of children’s endorsement (or not) for each word, their RT in endorsing (or not) each word and their recall (or not) of each word. As expected for typically developing children of late childhood, children endorsed a greater number of positive words (M/s.d. = 17.14/5.39) than negative words (M/s.d. = 4.81/4.77), *t*(113) = 15.17, *P* < 0.001.^1^ Children also showed a slower RT in responding to the positive words (M/s.d. = 979.57/799.86) than the negative words (M/s.d. = 843.46/532.36), *t*(113) = 2.46, *P* = 0.02. However, they recalled a similar number of positive (M/s.d. = 3.44/2.19) and negative words (M/s.d. = 3.63/2.20), *t*(113) = −0.75, *P* = 0.45.

### EEG data acquisition and processing

Children completed the SRET in an electrically shielded chamber, while continuous EEG signals were recorded via a 64-channel HydroCel GSN Electrical Geodesics Inc. (EGI) net and an EGI 200 NetAmps Amplifier (Electrical Geodesics Inc., Eugene, OR). Electrode impedances were kept <50 kΩ. EEG signals were recorded with a sampling rate of 250 Hz, referenced to the vertex electrode (Cz) during recording. EEG data were processed using the EEGLab (Delorme and Makeig, 2004) and ERPLab (Lopez-Calderon and Luck, 2014) toolboxes operated in MATLAB 9.10.0 (MathWorks, Inc., Natick, MA) and the Net Station Tools (Electrical Geodesics Inc., Eugene, OR).

Raw EEG data were first subjected to an offline filter (0.1–40 Hz) and re-referenced to the average of the two mastoid electrodes. Because no signal averaging was conducted to increase the signal-to-noise ratio, we used stringent artifact rejection methods to ensure a good quality of the trial-level data. First, we conducted an independent component analysis to detect and remove ocular artifacts. The pre-processed continuous EEG data were then time-locked to the onset of each word and segmented into epochs from 200 ms before to 1000 ms post the word onset, with a 200 ms baseline correction. We further rejected segments with (i) voltage beyond ±80 μV, (ii) a change of voltage >40 μV between timepoints or (iii) a change of voltage >250 μV between the most positive and most negative time points within a 200 ms moving window.

Out of the initial 115 children, we excluded 16 children without usable ERP data (15 had <10 accepted trials per condition, and one did not complete the SRET) and 3 children who did not complete the CDI, rendering a final sample size of 96 (nesting 4094 trials in total) for subsequent MLM analysis. Among the 96 participants, there was no difference in the number of accepted trials between the positive (mean/s.d. = 22.38/4.29) and negative (mean/s.d. = 22.27/4.64) conditions (*t*(95) = 0.40, *P* = 0.69).

### Quantification of ERP components

**Fig. 1.**
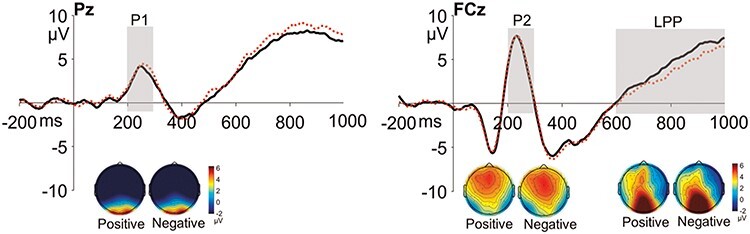
Grand average ERP waveforms and topographic maps of the P1, P2, aLPP and posterior components at Pz and FCz for the positive and negative SRET conditions.

We focused on several a priori ERP components elicited by the SRET, including the P1, P2 and LPP, which have been reported in the relevant literature. For the LPP component, although existing SRET studies have mostly reported an aLPP (e.g. Shestyuk and Deldin, 2010; Auerbach *et al*., 2015, 2016; Liu and Tan, 2023a,b), visual inspection of our ERP data showed a higher activity of the LPP in the posterior regions (see the topographic maps in Figure 1). Therefore, we quantified both the aLPP and the pLPP in the current study. The ERP components of interest were quantified in highly similar ways as those reported in the literature: the P1 and the pLPP were quantified as the mean amplitude across six parieto-occipital electrodes (Pz, P1, P2, POz, PO3 and PO4) for the post-stimulus time window of 100–200 ms and 500–1000 ms, respectively; the P2 and the aLPP were quantified as the mean amplitude across seven fronto-central electrodes (AFz, Fz, F1, F2, FCz, FC1 and FC2) for the 200–300 ms time window and the 600–1000 ms time window, respectively.

Figure 1 shows the grand average ERP waveforms and topographic maps of each ERP component. Paired-samples *t*-tests comparing the person-level averaged amplitude of each component between the positive and negative SRET conditions showed that a larger aLPP was elicited by the positive *vs* negative SRET words (*t*(95) = 2.52, *P* = 0.01); no differences were found for P1, P2 or pLPP (*P*s > 0.80).

For the MLM analysis, the trial-level amplitude of each ERP component (averaged across time points within the time window and the selected electrodes) was extracted from each accepted trial of each condition for each child. These trial-level ERP data were subjected to the MLM together with trial-level behavioral data: RT, endorsement/rejection of each word and recall (or fail to recall) of each word.

### MLM analysis

Trial-level repeated measures of the ERP and behavioral data of the SRET were organized into a nested structure (4094 trials nested within 96 children). We conducted three MLMs to accommodate this data structure and examine the extent to which trial-level ERPs predicted trial-level behavioral measures. All models treated the four ERP components (P1, P2, aLPP and pLPP) as independent variables (IVs). The behavioral measure of RT, endorsement and recall was treated as the dependent variable (DV) in each of the three models, respectively. The between-person level variables of SRET condition (negative = 0 and positive = 1), age, sex (girl = 0 and boy = 1) and CDI scores of depressive symptoms were entered as covariates. In the initial models, we also included the interaction terms between the SRET condition (positive *vs* negative) and each ERP component; none of the interactions was significant, and hence, they were dropped from the models to preserve power. Relationships among the extended set of variables were examined using two-level models via the lme4 package in R (version 1.1–29; Bates *et al*., 2015).

Equations (1a)–(5) illustrated Model 1 with RT as the DV. The repeated measures of RT for individual *i* at time *t*, ${\mathrm{Response\_Tim}}{{\mathrm{e}}_{it}}$, was modeled as a function of random intercepts, ${\beta _{oi}}$, that indicated the baseline level of RT. Among the coefficients, ${\beta _{1i}}$ to ${\beta _{4i}}$ indicated the within-person effects of the ERPs; ${\gamma _{00}}$ to ${\gamma _{08}}$ and ${\gamma _{10}}$ to ${\gamma _{40}}$ were the sample-level parameters; ${u_{0i}}$ were the between-person variations unexplained by the predictors and were assumed to be normally distributed with a mean of zero and variances of $\sigma _{{u_{0i}}}^2$. We did not include the random slopes lest the model become too complex to fit. Focusing on the fixed effects of interest was also consistent with the exploratory nature of this study.


(1a) $$\begin{aligned}{\mathrm{Response\_Tim}}{{\mathrm{e}}_{it}} =& {\beta _{oi}} + {\beta _{1i}} \times {\mathrm{wp}}.{\mathrm{P}}{1_{it}} + {\beta _{2i}} \times {\mathrm{wp}}.{\mathrm{P}}{2_{it}} + {\beta _{3i}} \nonumber \\ & \times {\mathrm{wp}}{\mathrm{.aLP}}{{\mathrm{P}}_{it}} + {\beta _{4i}} \times {\mathrm{wp}}{\mathrm{.pLP}}{{\mathrm{P}}_{it}} + {e_{it}}\end{aligned}$$

(2) $$\begin{aligned}
{\beta _{oi}} = \,& {\gamma _{00}} + {\gamma _{01}} \times bp.P{1_i} + + {\gamma _{02}} \times bp.P{2_i} + {\gamma _{03}} \times bp.aLP{P_i} \nonumber\\&
+ {\gamma _{04}} \times bp.pLP{P_i} + {\gamma _{05}} \times CD{I_i} + {\gamma _{06}} \times Ag{e_i} + {\gamma _{07}} \nonumber\\& \times Se{x_i} + + {\gamma _{08}} \times Conditio{n_i} + {\ }{u_{0i}}
\end{aligned}$$

(3) $${\beta _{1i}} = {\gamma _{10}}$$

(4) $${\beta _{2i}} = {\gamma _{20}}\\[-2pt]$$

(5) $${\beta _{3i}} = {\gamma _{30}}\\[-2pt]$$

(5) $${\beta _{4i}} = {\gamma _{40}}\\[4pt]$$

Unlike the RT, the other two DVs, endorsement and recall, were binary in nature on the trial level. To accommodate the binary DVs, we used generalized linear mixed-effects models and the link function of binomial distribution in Models 2 and 3. Equations (1b) and (1c) illustrated Model 2 with endorsement as the DV; the same equations applied to Model 3 with recall as the DV. In equation (1b), the DVs entered into the model were the log odds of the likelihood of endorsement (or recall). The specification of ${\beta _{oi}}$ to ${\beta _{3i}}$ was the same as that in equations (2)–(5). In equation (1c), the within-person residual term, $\varepsilon $, was not explicitly estimated; rather, it was assumed to follow a logistic distribution with a mean of zero, a scale of 1 and a variance of ${\ }\frac{{{\ }{\pi ^2}}}{3}$.


(1b) $$\begin{aligned}\log \left( {\frac{{\Pr \left( {Endorsemen{t_{it}}} \right)}}{{1 - \Pr \left( {Endorsemen{t_{it}}} \right)}}} \right) =& {\beta _{oi}} + {\beta _{1i}} \times wp.P{1_{it}} + {\beta _{2i}} \nonumber \\ & \times wp.P{2_{it}} + {\beta _{3i}} \times wp.aLP{P_{it}} \nonumber \\ & + {\beta _{4i}} \times wp.pLP{P_{it}}\end{aligned}$$

(1c) $$\begin{aligned}&Endorsemen{t_{it}} \nonumber \\ &= \frac{1}{{1 + {e^{ - \left( {{\beta _{oi}} + {\beta _{1i}} \times wp.P{1_{it}} + {\beta _{2i}} \times wp.P{2_{it}} + {\beta _{3i}} \times wp.aLP{P_{it}} + {\beta _{4i}} \times wp.pLP{P_{it}}} \right)}}}} + \varepsilon \end{aligned}$$

## Results

### Between-person descriptive statistics and bivariate correlations

Table 1 shows the between-person descriptive statistics and bivariate correlations of main study variables. Although not included in the MLM analysis, we also presented in Table 1 the positive and negative SRET scores (the number of positive or negative words both endorsed and recalled divided by the total number of words endorsed), which have been commonly used as indices of positive and negative self-referential processing biases in the literature (e.g. Shestyuk and Deldin, 2010; Auerbach *et al*., 2015, 2016; Liu and Tan, 2023a,b). As expected, higher CDI scores were associated with a lower positive SRET score and a higher negative SRET score. Positive and negative SRET scores were positively correlated with a higher overall recall rate. Higher CDI scores were correlated with a lower recall rate. A larger P1 was correlated with a larger pLPP. A larger P2 was correlated with a larger aLPP and longer RT.

**Table 1.** Between-person mean, standard deviation and bivariate correlations of main study variables

		1	2	3	4	5	6	7	8	9	10	11
1	Age (years)											
2	CDI	−0.00										
3	P1	0.04	0.08									
4	P2	−0.11	−0.05	0.18								
5	aLPP	0.00	0.08	0.16	0.44^***^							
6	pLPP	−0.13	0.00	0.29^**^	0.07	0.15						
7	RT (ms)	−0.07	0.15	0.08	0.29^**^	−0.01	−0.05					
8	Endorsement rate	−0.09	0.01	0.10	0.04	−0.09	−0.01	−0.11				
9	Recall rate	0.12	−0.27^**^	−0.14	−0.01	−0.11	−0.05	0.1−0.13	−0.09			
10	Positive SRET score	0.20	−0.47^**^	0.10	0.01	−0.01	−0.09	−0.00	−0.10	0.63^**^		
11	Negative SRET score	0.09	0.43^**^	−0.06	0.17	0.11	−0.02	0.14	0.04	0.29^**^	−0.14	
	Mean	11.05	8.25	0.93	4.74	3.09	5.05	795.98	0.49	0.16	0.13	0.03
	s.d.	1.46	8.27	3.81	4.58	6.10	5.05	370.94	0.09	0.07	0.08	0.05

*Note*:Endorsement rate: number of endorsed words/total number of words; recall rate: number of recalled words/total number of words; s.d.: standard deviation.

** *P* < 0.01,

*** *P* < 0.001.

### Associations between the ERP and behavioral measures of the SRET

Table 2 presents the results of the three MLMs. In Model 1 with the RT as the DV, the within-person effect of P1 was significant (${\gamma _{10}}$ = 3.61, *P *= 0.003) such that for a child, a larger P1 on a given trial (relative to this child’s mean P1) predicted a longer RT in responding to this trial. The within-person effects of P2 (${\gamma _{20}}$ = −3.12, *P *< 0.001) and pLPP (${\gamma _{40}}$ = −4.34, *P *< 0.001) were significant on the RT, such that for a child, a larger P2 or a larger pLPP on a given trial (compared to this child’s mean P2 or pLPP) predicted a shorter RT in responding to this trial. The between-person effect of P2 on the RT was also significant (${\gamma _{02}}$ = 29.47, *P *= 0.002), such that children with a larger mean P2 (relative to other children) showed a longer RT in general. The between-person effect of condition was significant, with the RT for the positive words longer than that for the negative words (${\gamma _{08}}$ = 49.04, *P* = 0.03). None of the other covariates (age, sex and depressive symptoms) showed a significant effect.

**Table 2.** Results of the three MLMs with P1, P2, aLPP and pLPP as the IVs

	Model 1: DV = RT			Model 2: DV = endorsement			Model 3: DV = recall		
Fixed effects	Estimate	*SE*	*P*	Estimate	*SE*	*P*	Estimate	*SE*	*P*
Intercept, ${\gamma _{00}}$	775.10	50.74	**<0.001**	−1.28	0.09	**<0.001**	−1.63	0.08	**<0.001**
wp.P1, ${{\gamma}_{10}}$	3.61	1.22	**0.003**	0.003	0.004	0.52	−0.002	0.005	0.75
wp.P2, ${\gamma _{20}}$	−3.12	0.91	**<0.001**	−0.003	0.003	0.42	0.004	0.004	0.26
wp.aLPP, ${\gamma _{30}}$	1.52	0.85	0.07	0.006	0.003	**0.03**	0.0006	0.003	0.87
wp.pLPP, ${\gamma _{40}}$	−4.34	0.81	**<0.001**	0.001	0.003	0.65	0.002	0.004	0.55
bp.P1, ${\gamma _{01}}$	4.22	10.94	0.70	0.01	0.02	0.43	−0.022	0.02	0.18
bp.P2, ${\gamma _{02}}$	29.47	9.19	**0.002**	0.01	0.01	0.46	0.009	0.014	0.53
bp.aLPP, ${\gamma _{03}}$	−10.64	6.71	0.12	−0.02	0.01	0.13	−0.01	0.01	0.30
bp.pLPP, ${\gamma _{04}}$	−4.95	8.20	0.55	−0.002	0.01	0.89	0.004	0.012	0.74
bp.CDI, ${\gamma _{05}}$	7.42	4.63	0.11	−0.002	0.007	0.82	−0.02	0.007	**0.005**
bp.Age, ${\gamma _{06}}$	−11.46	25.70	0.67	−0.03	0.04	0.42	0.05	0.04	0.20
bp.Sex, ${\gamma _{07}}$	−20.79	85.06	0.81	−0.15	0.13	0.25	−0.12	0.13	0.34
bp.Condition, ${\gamma _{08}}$	49.04	21.87	**0.03**	2.51	0.08	**<0.001**	−0.07	0.09	0.44

*Note: SE* = standard error; Sex: 1 = boy and 0 = girl; Condition: 1 = positive and 0 = negative; wp.: within-person effect; bp.: between-person effect; bold font: *p* < .05.

In Model 2 with the endorsement of words as the DV, the within-person effect of the aLPP was significant (${\gamma _{30}}$ = 0.006, *P *= 0.03); for a child, a larger aLPP on a given trial (relative to this child’s mean aLPP) was associated with a higher likelihood of endorsing the word in this trial. The between-person effect of aLPP was not significant. No significant effect, within-person or between-person, was found for P1 or P2. The between-person effect of condition was significant, with the likelihood of endorsing positive words higher than that of negative words (${\gamma _{08}}$ = 2.51, *P *< 0.001). No other covariate was associated with the word endorsement. In Model 3 with the recall of words as the DV, none of the within-person or between-person effects of the ERPs was significant. Among the covariates, higher depressive symptoms were associated with a lower likelihood of recall (${\gamma _{05}}$ = −0.02, *P* = 0.005). No other covariate showed a significant effect.

## Discussion

Among the vastness of socio-emotional information, self-referential information is particularly unique and salient even for children. The literature has used the SRET paradigm, sometimes combined with the ERPs, to study self-referential processing and its associations with psychopathology in youths. However, it is unclear how the brain and behavioral indices of self-referential processing are associated with each other, although this knowledge can inform our mechanistic understanding of this construct and its role in psychopathology. This study examined this question in a group of typically developing 9- to 12-year-old children, a critical period for the development of self-related concepts. Instead of between-person analysis, we used a novel MLM approach to disaggregate the between- and within-person sources of variability. As expected, we observed differential patterns of the ERP-behavior associations on the within- *vs* between-person levels (Table 2). Our study provided novel evidence on the extent to which the within-person variability of the ERP indices of self-referential processing predicted the overt responses of the SRET in children. These findings will improve our mechanistic knowledge of self-referential processing and lay the groundwork to a better understanding of the role of self-referential processing in the development of psychopathology.

In Model 1 with RT as the DV, we found that a larger P1 predicted a longer RT on the within-person level. For a child, if a given word elicited a greater P1 (compared to the mean P1 of this child), this child tended to respond slower in endorsing (or rejecting) this word, whether the word was positive or negative. The P1 reflects early, fast visual attentional processing based on lower-level stimulus features (Zhang & Luck, 2009), perceived task demand (Taylor, 2002) or (negative) emotional arousal (Luo *et al*., 2010; Zhang *et al*., 2014). In this context, an enhanced trial-level P1 suggested that the child might perceive the given trial as more demanding or the given personal trait word as more (negatively) arousing (regardless of the word valence; e.g. they might find some of the positive words negatively arousing when the word described a trait they wanted to have but did not possess). These very early perceptions of the given trial might be irrelevant to the task goal and have hampered the child’s speed in responding to the word.

We found a similar pattern in the within-person effects of the P2 and pLPP on the RT, such that a larger P2 or a larger pLPP predicted a shorter RT on the within-person level. For a child, if a given word elicited a greater P2 or a greater pLPP (compared to the mean of this child), this child tended to respond faster in endorsing or rejecting this word regardless of the word valence. This observation was consistent with previous findings in adults, which reported that on the within-person level, a larger face-elicited P2 predicted faster responses in categorizing the gender or race of this face (Volpert-Esmond and Bartholow, 2021). Unlike the P1 that is driven by lower-level stimulus features such as arousal, the visual P2 reflects more controlled, goal-oriented attentional processes such as attention allocation and mobilization toward goal-relevant cues (Schupp *et al*., 2004; Bar-Haim *et al*., 2005; Eldar *et al*., 2010; Volpert-Esmond and Bartholow, 2019). For a child performing the SRET, a larger P2 elicited by a given word may reflect more efficient extraction of goal-relevant information and greater goal-oriented attentional allocation toward this word, which accelerated the child’s subsequent response in deciding whether this word was self-descriptive.

The pLPP reflects deep, elaborative processing of socio-emotionally salient information that involves sustained engagement and memory retrieval (Cuthbert *et al*., 2000; Foti and Hajcak, 2008; Foti *et al*., 2009; Macnamara *et al*., 2009; Weinberg *et al*., 2012, 2021). The within-person effect of the pLPP on the RT suggested that for a child, when a given word elicited a more elaborative or more sustained processing compared to other words (indexed by a larger pLPP), the child responded faster to this word relative to other words. Our findings of the within-person effects of the P1, P2 and pLPP on the RT extended the existing literature in adults (Volpert-Esmond and Bartholow, 2021), suggesting that the within-person relationship between ERPs and RT was evident across different cognitive processes and different populations, including self-referential processing in children.

In predicting the RT on the ‘between-person’ level, the effect of P2 was in the opposite direction to the within-person effect of P2 (i.e. a larger P2 predicted a longer RT on the between-person level), whereas the effect of the P1 or the pLPP was non-significant. This between-person P2-RT relationship was not reported in previous studies (Dickter and Bartholow, 2007; Kubota and Ito, 2007; Volpert-Esmond and Bartholow, 2021). The between-person effect represents the time-invarying portion of variability, i.e. the effect of trait-like characteristics that differ from person to person but relatively stable over time (Curran and Bauer, 2011). In our data, the absence of the between-person effects of P1 and pLPP suggested that children with a trait-like, consistently larger (or smaller) P1 or pLPP relative to other children did not necessarily respond faster (or slower) than others. However, it is unclear why children with a trait-like, larger P2 relative to other children showed slower responses. One possibility was that for an individual, a trait-like larger (or smaller) ERP component across trials might not reflect cognitive processing but rather was associated with other between-person factors such as neuroanatomical characteristics (e.g. the thickness of the skull; Chauveau *et al*., 2004; Hagemann *et al*., 2008). These between-person factors might have systematically contributed to (and potentially confounded) the observed between-person effect of P2.

In Model 2, the within-person effect of the aLPP on endorsement was significant: for a child, a larger aLPP elicited by a given word (compared to the mean aLPP of this child) predicted a higher likelihood of endorsing this word regardless of the word valence. As discussed earlier, some SRET studies, including our recent work, have observed an enhanced aLPP toward positive *vs* negative words (e.g. Auerbach *et al*., 2016; Liu and Tan, 2023b), whereas the pLPP did not differ between the two conditions (Liu and Tan, 2023b). Using the same dataset as the one in the current study, we also found that a smaller aLPP toward positive SRET words predicted depressive symptoms beyond the behavioral responses of the SRET in children, whereas the pLPP showed a much weaker association with depressive symptoms (Liu and Tan, 2023b). These differential patterns between the aLPP and pLPP suggested that the SRET-elicited aLPP might reflect the in-depth processing of the more ‘self-specific’ aspect of information, while the SRET-elicited pLPP might reflect the processing of the more ‘generic’ aspect of the information.

As a result, for a child performing the SRET, when a given word elicited a more elaborative ‘self-specific’ processing compared to other words (indexed by a larger aLPP), the child was more likely to endorse this word as ‘self-descriptive’. A larger trial-level aLPP may also indicate that the child successfully retrieved autobiographic memories relevant to this given word and, as a result, was more likely to endorse the word as describing themselves. One the other hand, the between-person effect of the aLPP was not significant in predicting endorsement, suggesting that the children with a trait-like larger aLPP relative to other children did not necessarily endorse more words as self-descriptive. Rather, it might be associated with other between-person factors such as neuroanatomical characteristics (Chauveau *et al*., 2004; Hagemann *et al*., 2008).

Model 3 with recall as the DV did not yield any significant effects of the ERPs. This was contrary to our expectation that the LPPs, which potentially involve effortful memory retrieval, might predict the recall of the SRET words. We also did not replicate the between-person level association between the aLPP and recall found in healthy adolescents of ages 13–18 years (Auerbach *et al*., 2016). One factor related to the absence of results might be that in our task, 60 words for an unexpected recall task were too much for 9- to 12-year-old children. Indeed, our children recalled only 7 out of the 60 words on average (s.d. = 3.67, ranging 0–20).

It is interesting to note that the ERP components predicted different aspects of the behavioral response on the within-person level, with the P1, P2 and pLPP associated with the RT and the aLPP associated with the categorical decision (i.e. endorse or not). Relating back to the continuous flow model of information processing (Coles *et al*., 1985, 1995), we speculate that there may exist more than one path of flow, partially in parallel, with different stages of real-time information processing (e.g. earlier sensory/attentional processing or later elaborate processing) contributing to distinct facets of the behavioral outputs. Further, these different paths of information flow may show differential associations with the development of psychopathology, warranting future investigations of this topic within an MLM framework.

One limitation of this study is that in data analysis, we did not differentiate between trials with words that were endorsed *vs* not endorsed. Although endorsed words and non-endorsed words were likely to elicit highly similar cognitive processes, it would be interesting to directly compare these two conditions. Considering the low endorsement rate of negative words in typically developing youths, future research using a larger number of words (especially negative ones) is needed to generate sufficient usable trials to examine the endorsed (*vs* non-endorsed) words. The unexpected free recall task we used might be too difficult for 9- to 12-year-old children, generating low recall rate with limited individual variation. Future research using a relatively easier memory task (e.g. a recognition task) may generate improved performance and better speak to the LPP-memory relationship. Future studies will also benefit from a larger sample size, which will be useful in isolating the confounding between-person factors (e.g. neuroanatomical characteristics) from effects of interest.

Regardless of these limitations, we observed interesting patterns of the within-person level associations between the ERP correlates and behavioral outputs of self-referential processing, including associations between a larger P1 and a longer RT, a larger P2 and a shorter RT, a larger pLPP and a shorter RT and a larger aLPP and a higher likelihood of self-endorsement. Although this study was still largely exploratory, we provided the first evidence of the within-person level brain-behavior relationships in the context of children’s self-referential processing, a unique aspect of socio-emotional processing that is known to play an important role in child development and psychopathology. Along this line, we will investigate the associations between self-referential processing and psychopathology within an MLM framework in the next step of our research.

## Data Availability

The data that support the findings of this study are available from the corresponding author, P.L., upon reasonable request.

## Footnotes

1 Out of the 115 children who participated in this study, 114 of them completed the SRET and contributed valid behavioral data.

## References

[R1] Allison G.O., Benau E.M., Asbaghi S., Pagliacco D., Stewart J.G., Auerbach R.P. (2021). Neurophysiological markers related to negative self-referential processing differentiate adolescent suicide ideators and attempters. *Biological Psychiatry Global Open Science*, 1(1), 16–27. 36324429 10.1016/j.bpsgos.2021.04.001 PMC9616352

[R2] Andrews G., Murphy K., Dunbar M. (2020). Self-referent encoding facilitates memory binding in young children: New insights into the self-reference effect in memory development. *Journal of Experimental Child Psychology*, 198, 104919. 10.1016/j.jecp.2020.104919 32629234

[R3] Auerbach R.P., Bondy E., Stanton C.H., Webb C.A., Shankman S.A., Pizzagalli D.A. (2016). Self-referential processing in adolescents: stability of behavioral and ERP markers. *Psychophysiology*, 53(9), 1398–406. 27302282 10.1111/psyp.12686 PMC4982841

[R4] Auerbach R.P., Stanton C.H., Proudfit G.H., Pizzagalli D.A. (2015). Self-referential processing in depressed adolescents: a high-density event-related potential study. *Journal of Abnormal Psychology*, 124(2), 233–45. 25643205 10.1037/abn0000023 PMC4429006

[R5] Bar-Haim Y., Lamy D., Glickman S. (2005). Attentional bias in anxiety: a behavioral and ERP study. *Brain and Cognition*, 59(1), 11–22. 15919145 10.1016/j.bandc.2005.03.005

[R6] Bates D., Mächler M., Bolker B., Walker S. (2015). Fitting linear mixed-effects models using lme4. *Journal of Statistical Software*, 67(1), 1–48.

[R7] Bradley M.M., Lang P.J. (1999). Affective Norms for English Words (ANEW): stimuli instruction, and affective ratings (*Tech. Rep. No. C-1*). Gainesville, FL: University of Florida, Center for Research in Psychophysiology.

[R8] Cassidy S.M., Robertson I.H., O’Connell R.G. (2012). Retest reliability of event-related potentials: evidence from a variety of paradigms. *Psychophysiology*, 49(5), 659–64. 22335452 10.1111/j.1469-8986.2011.01349.x

[R9] Chauveau N., Franceries X., Doyon B., Rigaud B., Morucci J.P., Celsis P. (2004). Effects of skull thickness, anisotropy, and inhomogeneity on forward EEG/ERP computations using a spherical three-dimensional resistor mesh model. *Human Brain Mapping*, 21(2), 86–97. 14755596 10.1002/hbm.10152 PMC6872130

[R10] Coles M.G., Gratton G., Bashore T.R., Eriksen C.W., Donchin E. (1985). A psychophysiological investigation of the continuous flow model of human information processing. *Journal of Experimental Psychology: Human Perception and Performance*, 11(5), 529–53. 2932529 10.1037//0096-1523.11.5.529

[R11] Coles M.G.H. Smid H.G.O.M. Scheffers M.K. Otten L.J. (1995). Mental chronometry and the study of human information processing. In: Rugg, M.D., Coles, M.G.H. editors. *Electrophysiology of Mind: Event-related Brain Potentials and Cognition*, pp. 86–131, Oxford, United Kingdom: Oxford University Press.

[R12] Crowley K.E., Colrain I.M. (2004). A review of the evidence for P2 being an independent component process: age, sleep and modality. *Clinical Neurophysiology: Official Journal of the International Federation of Clinical Neurophysiology*, 115(4), 732–44. 15003751 10.1016/j.clinph.2003.11.021

[R13] Curran P.J., Bauer D.J. (2011). The disaggregation of within-person and between-person effects in longitudinal models of change. *Annual Review of Psychology*, 62, 583–619. 10.1146/annurev.psych.093008.100356 PMC3059070 19575624

[R0014a] Cuthbert B.N., Insel T.R. (2013). Toward the future of psychiatric diagnosis: the seven pillars of RDoC. *BMC Medicine*, 11, 126. 10.1186/1741-7015-11-126 PMC3653747 23672542

[R14] Cuthbert B.N., Schupp H.T., Bradley M.M., Birbaumer N., Lang P.J. (2000). Brain potentials in affective picture processing: covariation with autonomic arousal and affective report. *Biological Psychology*, 52(2), 95–111. 10699350 10.1016/s0301-0511(99)00044-7

[R15] Delorme A., Makeig S. (2004). EEGLAB: an open source toolbox for analysis of single-trial EEG dynamics including independent component analysis. *Journal of Neuroscience Methods*, 134(1), 9–21. 15102499 10.1016/j.jneumeth.2003.10.009

[R16] Derry P.A., Kuiper N.A. (1981). Schematic processing and self-reference in clinical depression. *Journal of Abnormal Psychology*, 90(4), 286–97. 7264058 10.1037//0021-843x.90.4.286

[R17] Dickter C.L., Bartholow B.D. (2007). Racial ingroup and outgroup attention biases revealed by event-related brain potentials. *Social Cognitive & Affective Neuroscience*, 2(3), 189–98. 18985140 10.1093/scan/nsm012 PMC2569810

[R18] Eldar S., Yankelevitch R., Lamy D., Bar-Haim Y. (2010). Enhanced neural reactivity and selective attention to threat in anxiety. *Biological Psychology*, 85(2), 252–7. 20655976 10.1016/j.biopsycho.2010.07.010

[R19] Foti D., Hajcak G. (2008). Deconstructing reappraisal: descriptions preceding arousing pictures modulate the subsequent neural response. *Journal of Cognitive Neuroscience*, 20(6), 977–88. 18211235 10.1162/jocn.2008.20066

[R20] Foti D., Hajcak G., Dien J. (2009). Differentiating neural responses to emotional pictures: evidence from temporal-spatial PCA. *Psychophysiology*, 46(3), 521–30. 19496228 10.1111/j.1469-8986.2009.00796.x

[R21] Goldstein B.L., Hayden E.P., Klein D.N. (2015). Stability of self-referent encoding task performance and associations with change in depressive symptoms from early to middle childhood. *Cognition and Emotion*, 29(8), 1445–55. 25530070 10.1080/02699931.2014.990358 PMC4476963

[R22] Hagemann D., Hewig J., Walter C., Naumann E. (2008). Skull thickness and magnitude of EEG alpha activity. *Clinical Neurophysiology: Official Journal of the International Federation of Clinical Neurophysiology*, 119(6), 1271–80. 18387340 10.1016/j.clinph.2008.02.010

[R23] Hillyard S.A., Vogel E.K., Luck S.J., Humphreys G.W., Duncan J., Treisman A. (1998). Sensory gain control (amplification) as a mechanism of selective attention: electrophysiological and neuroimaging evidence. *Philosophical Transactions of the Royal Society of London Series B, Biological Sciences*, 353(1373), 1257–70. 9770220 10.1098/rstb.1998.0281 PMC1692341

[R24] Hudson A., Green E.S., Wilson M.J.G., Itier R.J., Henderson H.A. (2021). The prominence of self-referential processing across ERP and memory consolidation in children. *Developmental Neuropsychology*, 46(8), 598–615. 34696639 10.1080/87565641.2021.1991354

[R25] Huffmeijer R., Bakermans-Kranenburg M.J., Alink L.R.A., van Ijzendoorn M.H. (2014). Reliability of event-related potentials: the influence of number of trials and electrodes. *Physiology and Behavior*, 130, 13–22. 24642000 10.1016/j.physbeh.2014.03.008

[R0026a] Kovacs M. (1978). Children’s Depression Inventory (CDI) [Database record]. *APA PsycTests*.

[R26] Kubota J.T., Ito T.A. (2007). Multiple cues in social perception: the time course of processing race and facial expression. *Journal of Experimental Social Psychology*, 43(5), 738–52. 17940587 10.1016/j.jesp.2006.10.023 PMC2031842

[R27] Kuiper N.A., Derry P.A. (1982). Depressed and nondepressed content self-reference in mild depressives. *Journal of Personality*, 50(1), 67–80. 7086630 10.1111/j.1467-6494.1982.tb00746.x

[R28] Liu P., Tan J.X.Y. (2023a). Behavioral and ERP indices of self-schematic processing show differential associations with emerging symptoms of depression and social anxiety in late childhood: evidence from a community-dwelling sample. *Biological Psychology*, 180, 108594. 10.1016/j.biopsycho.2023.108594 PMC10357463 37247814

[R29] Liu P., Tan J.X.Y. (2023b). Incremental validity of ERP correlates of self-referential processing in predicting emerging depressive symptoms in late childhood: evidence from a community-dwelling sample. *Psychophysiology*, 60(11), e14382. 10.1111/psyp.14382 37392027

[R30] Liu P., Vandermeer M.R.J., Joanisse M.F., Barch D.M., Dozois D.J.A., Hayden E.P. (2020a). Depressogenic self-schemas are associated with smaller regional grey matter volume in never-depressed preadolescents. *NeuroImage: Clinical*, 28, 102422. 10.1016/j.nicl.2020.102422 PMC7502366 32949875

[R31] Liu P., Vandermeer M.R.J., Joanisse M.F., Barch D.M., Dozois D.J.A., Hayden E.P. (2020b). Neural activity during self-referential processing in children at risk for depression. *Biological Psychiatry: Cognitive Neuroscience and Neuroimaging*, 5(4), 429–37. 32081615 10.1016/j.bpsc.2019.12.012

[R0031a] Luo W., Feng W., He W., Wang N.-Y., Luo Y.-J. (2010). Three stages of facial expression processing: ERP study with rapid serial visual presentation. *NeuroImage*, 49(2), 1857–67. 19770052 10.1016/j.neuroimage.2009.09.018 PMC3794431

[R32] Lopez-Calderon J., Luck S.J. (2014). ERPLAB: an open-source toolbox for the analysis of event-related potentials. *Frontiers in Human Neuroscience*, 8, 213. 10.3389/fnhum.2014.00213 PMC3995046 24782741

[R33] Luck S.J. (2014). *An Introduction to the Event-related Potential Technique*. Cambridge, Massachusetts: MIT press.

[R34] Macnamara A., Foti D., Hajcak G. (2009). Tell me about it: neural activity elicited by emotional pictures and preceding descriptions. *Emotion (Washington, D.C.)*, 9(4), 531–43. 19653776 10.1037/a0016251

[R0035a] Mangun G.R., Buck L.A. (1998). Sustained visual-spatial attention produces costs and benefits in response time and evoked neural activity. *Neuropsychologia*, 36(3), 189–200. 9622184 10.1016/s0028-3932(97)00123-1

[R35] Mangun G.R. (1995). Neural mechanisms of visual selective attention. *Psychophysiology*, 32(1), 4–18. 7878167 10.1111/j.1469-8986.1995.tb03400.x

[R36] Mills K.L., Siegmund K.D., Tamnes C.K., et al. (2021). Inter-individual variability in structural brain development from late childhood to young adulthood. *NeuroImage*, 242, 118450. 10.1016/j.neuroimage.2021.118450 PMC8489572 34358656

[R37] Molenaar P.C.M. (2004). A manifesto on psychology as idiographic science: bringing the person back into scientific psychology, this time forever. *Measurement: Interdisciplinary Research and Perspectives*, 2(4), 201–18.

[R38] Northoff G., Heinzel A., de Greck M., Bermpohl F., Dobrowolny H., Panksepp J. (2006). Self-referential processing in our brain-A meta-analysis of imaging studies on the self. *NeuroImage*, 31(1), 440–57. 16466680 10.1016/j.neuroimage.2005.12.002

[R39] Pfeifer J.H. Dapretto M. Lieberman M.D. (2016). The neural foundations of evaluative self-knowledge in middle childhood, early adolescence, and adulthood. In: Zelazo, P.D., Chandler, M., Crone, E., editors. *Developmental Social Cognitive Neuroscience*, pp. 155–78, London, England: Psychology Press.

[R40] Pfeifer J.H., Lieberman M.D., Dapretto M. (2007). “I know you are but what am I?!” Neural bases of self- and social knowledge retrieval in children and adults. *Journal of Cognitive Neuroscience*, 19(8), 1323–37. 17651006 10.1162/jocn.2007.19.8.1323 PMC3407805

[R41] Regtvoort A.G.F.M., van Leeuwen T.H., Stoel R.D., van der Leij A. (2006). Efficiency of visual information processing in children at-risk for dyslexia: habituation of single-trial ERPs. *Brain and Language*, 98(3), 319–31. 16870246 10.1016/j.bandl.2006.06.006

[R42] Schupp H.T., Öhman A., Junghöfer M., Weike A.I., Stockburger J., Hamm A.O. (2004). The facilitated processing of threatening faces: an ERP analysis. *Emotion*, 4(2), 189–200. 15222855 10.1037/1528-3542.4.2.189

[R43] Shestyuk A.Y., Deldin P.J. (2010). Automatic and strategic representation of the self in major depression: trait and state abnormalities. *The American Journal of Psychiatry*, 167(5), 536–44. 20360316 10.1176/appi.ajp.2009.06091444

[R44] Speed B.C., Nelson B.D., Auerbach R.P., Klein D.N., Hajcak G. (2016). Depression risk and electrocortical reactivity during self-referential emotional processing in 8 to 14 year-old girls. *Journal of Abnormal Psychology*, 125(5), 607–19. 27175985 10.1037/abn0000173 PMC4925302

[R0040a] Taylor M.J. (2002). Non-spatial attentional effects on P1. *Clinical Neurophysiology: Official Journal of the International Federation of Clinical Neurophysiology*, 113(12), 1903–8. 12464327 10.1016/s1388-2457(02)00309-7

[R45] Volpert-Esmond H.I., Bartholow B.D. (2019). Explicit categorization goals affect attention-related processing of race and gender during person construal. *Journal of Experimental Social Psychology*, 85, 103839. 10.1016/j.jesp.2019.103839 PMC7442203 32831396

[R46] Volpert-Esmond H.I., Bartholow B.D. (2021). A functional coupling of brain and behavior during social categorization of faces. *Personality and Social Psychology Bulletin*, 47(11), 1580–95. 33419384 10.1177/0146167220976688 PMC8263806

[R47] Volpert-Esmond H.I., Merkle E.C., Levsen M.P., Ito T.A., Bartholow B.D. (2018). Using trial-level data and multilevel modeling to investigate within-task change in event-related potentials. *Psychophysiology*, 55(5), e13044. 10.1111/psyp.13044 PMC5899682 29226966

[R48] Volpert-Esmond H.I., Page-Gould E., Bartholow B.D. (2021). Using multilevel models for the analysis of event-related potentials. *International Journal of Psychophysiology: Official Journal of the International Organization of Psychophysiology*, 162, 145–56. 33600841 10.1016/j.ijpsycho.2021.02.006 PMC8050933

[R49] Von Gunten C.D., Volpert-Esmond H.I., Bartholow B.D. (2018). Temporal dynamics of reactive cognitive control as revealed by event-related brain potentials. *Psychophysiology*, 55(3). 10.1111/psyp.13007 PMC5811320 28960342

[R50] Weinberg A., Correa K.A., Stevens E.S., Shankman S.A. (2021). The emotion-elicited late positive potential is stable across five testing sessions. *Psychophysiology*, 58(11), e13904. 10.1111/psyp.13904 34292629

[R51] Weinberg A., Hilgard J., Bartholow B.D., Hajcak G. (2012). Emotional targets: evaluative categorization as a function of context and content. *International Journal of Psychophysiology: Official Journal of the International Organization of Psychophysiology*, 84(2), 149–54. 22342564 10.1016/j.ijpsycho.2012.01.023

[R52] Wise S., Huang-Pollock C., Pérez-Edgar K. (2022). Implementation of the diffusion model on dot-probe task performance in children with behavioral inhibition. *Psychological Research*, 86(3), 831–43. 34047824 10.1007/s00426-021-01532-3 PMC8627521

[R53] Woestenburg J.C., Verbaten M.N., Van Hees H.H., Slangen J.L. (1983). Single trial ERP estimation in the frequency domain using orthogonal polynomial trend analysis (OPTA): estimation of individual habituation. *Biological Psychology*, 17(2–3), 173–91. 6640015 10.1016/0301-0511(83)90018-2

[R0030a] Zhang D., He W., Wang T., et al. (2014). Three stages of emotional word processing: an ERP study with rapid serial visual presentation. *Social Cognitive and Affective Neuroscience*, 9(12), 1897–1903. 24526185 10.1093/scan/nst188 PMC4249467

[R0053a] Zhang W., Luck S.J. (2009). Feature-based attention modulates feedforward visual processing. *Nature Neuroscience*, 12(1), 24–25. 19029890 10.1038/nn.2223

